# Regularities of Microstructure Evolution in a Cu-Cr-Zr Alloy during Severe Plastic Deformation

**DOI:** 10.3390/ma15165745

**Published:** 2022-08-20

**Authors:** Anna Bodyakova, Maksim Tkachev, Georgy I. Raab, Rustam Kaibyshev, Andrey N. Belyakov

**Affiliations:** 1 Laboratory of Mechanical Properties of Nanostructured Materials and Superalloys, Belgorod State University, Belgorod 308015, Russia; 2 Magnitogorsk State Technical University, Magnitogorsk 455000, Russia; 3 Laboratory of Prospective Steels for Agricultural Machinery, Russian State Agrarian University, Moscow Timiryazev Agricultural Academy, Moscow 127550, Russia

**Keywords:** copper alloy, severe plastic deformation, grain refinement, strengthening

## Abstract

The effect of severe plastic deformation by the conforming process of equal channel angular extrusion (ECAE-Conform) followed by cold rolling on the microstructures developed in a Cu-0.1Cr-0.1Zr alloy was investigated. Following the ECAE-Conform of 1 to 8 passes (corresponding strains were 0.8 to 6.4) cold rolling to a total strain of 4 was accompanied by substantial grain refinement and strengthening. An average grain size tended to approach 160 nm with an increase in the rolling reduction. An increase in the ECAE-Conform strain promoted the grain refinement during subsequent cold rolling. The fraction of the ultrafine grains with a size of 160 nm after cold rolling to a strain of 4 increased from 0.12 to 0.52 as the number of ECAE-Conform passes increased from 1 to 8. Correspondingly, the yield strength increased above 550 MPa. The strengthening could be expressed by a Hall–Petch type relationship with a grain size strengthening factor of 0.11 MPa m^0.5^.

## 1. Introduction

A significant breakthrough in recent years in the field of new materials and processing methods is associated with the development of severe plastic deformation (SPD), which can be used to obtain new materials with multifunctional properties [1,2]. In particular, SPD is expected to result in an optimal microstructural design providing high strength. The latter is important for conductive materials that require high strength along with electro-conductivity [3,4,5]. The promising microstructures of copper and its alloys with high strength and conductivity involve the ultrafine grains (UFG) containing dispersed nanosized particles [3,4,5,6,7,8,9]. The UFG microstructure with a high dislocation density increases the strength by a factor of 2–3 with a slight decrease in electrical conductivity by 5–10% IACS [3,4,5,6,7,8,9,10,11]. Dispersed particles are effective obstacles to the movement of grain boundaries and dislocations, providing thermal stability of the material and strengthening it [12,13,14,15,16]. On the other hand, the particle precipitation removes the solutes from the copper matrix and increases the electrical conductivity of the alloy [17,18]. The dispersion strengthened copper alloys are alloyed by elements having a low equilibrium concentration at room temperature and the highest possible solubility at temperatures close to the melting point of copper. Those are Cr, Zr, Hf, Ti, Ag, etc. [19,20,21,22,23,24,25,26]. Recently, much attention has been paid to alloys with Cr and Zr that could be strengthened by 100–150 MPa with a little effect on their conductivity [3,4].

High-pressure torsion (HPT) is an effective SPD method [3,9,14,27,28]. HPT for one revolution at room temperature may produce an UFG microstructure in small cylindrical specimens of various copper alloys [3,9,14,29]. The main disadvantage of HPT is the difficulty of scaling it up for industrial applications. Another effective SPD method is equal channel angular extrusion/pressing (ECAE/P) [30,31,32,33,34]. The ECAE modification using the Conform scheme ensures the production of long billets for the manufacturing of wire rods/wires [35,36]. ECAE-Conform results in the grain refinement and makes it possible to obtain long billets with a UFG microstructure. The development of an equiaxed UFG microstructure during ECAE is associated with the formation of a 3D network of new strain-induced grain boundaries as a result of the grain subdivision owing to the action of different slip systems within one grain [32,33]. Thus, the new grain evolution results from a kind of continuous strain-induced reaction and can be considered as a continuous dynamic recrystallization (DRX), which consists in the formation of low-angle boundaries (LAB) followed by an increase in their misorientations and transformation to high-angle boundaries (HAB) with straining. The grain size achievable by continuous DRX is controlled by the size of subgrains evolved at earlier stages of deformation [4,37,38]. The size of deformation grains and subgrains depends on the temperature/strain rate conditions. A decrease in deformation temperature leads to a decrease in the size of the formed crystallites [38,39,40]. The grain size of 300 nm can be attained in low-alloy copper alloys by ECAE at room temperature [3]. The dislocation density also tends to saturate after strains of 2–4 [4,37,38], which can be explained in accordance with the Taylor model by the relation between the subgrain size and the dislocation density [41]. As a result, stabilization of the strength properties is observed [3,4].

It can be assumed that additional grain refinement can be achieved by changing the deformation scheme. A combination of ECAE-Conform with other methods of plastic deformation may be quite beneficial. Rolling is one of the industrially significant methods of plastic deformation and has proven to be an effective one for the hardening of copper alloys [42,43,44,45,46]. The formation of a sharp texture during rolling makes it possible to implement an additional textural strengthening [47]. However, studies devoted to the effect of combined treatments on the microstructure and properties of copper alloys are poorly covered in the literature. Kapoor et al. showed that rolling led to pronounced grain refinement, increasing the dislocation density and hardening of a copper alloy [48]. Yuan et al. studied the effect of one ECAE-Conform and extrusion on the microstructure and properties of copper alloys [49,50,51,52]. Asfandiyarov et al. reported about the implementation of the ECAP-Conform process and extrusion for Cu-Cr alloys in one installation [53]. Processing ensures the achievement of high strength up to 550 MPa with an electrical conductivity of 70–75% IACS. However, the distribution of temperature and strain over the cross section of the sample subjected to ECAE-Conform and rolling was not uniform [54]. The development of a homogeneous microstructure can be promoted by increasing the number of ECAE passes before cold rolling. Moreover, an increase in the number of ECAE passes should strengthen the copper alloy owing to grain refinement. Therefore, the purpose of this work is to study the microstructural changes during cold rolling of a Cu-Cr-Zr alloy subjected to ECAE-Conform to different total strains and to reveal the effect of the developed microstructures on the mechanical properties. It should be noted that the selected object of the investigation is of a great practical importance. The studied combination of ECAE-Conform and cold rolling is very promising processing method, because it can be easily scaled up to commercial production. Elaborating the regularities of the microstructure evolution and properties of an advanced material subjected to ECAE-Conform followed by cold rolling allows us solving the problem of producing the innovative materials.

## 2. Materials and Methods

**Figure 1:**
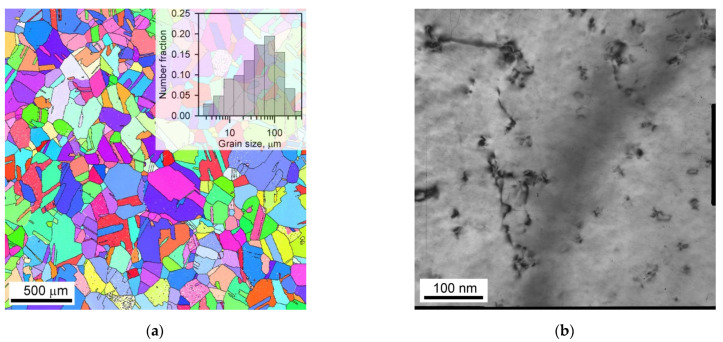
Initial microstructure (**a**) and dispersed particles (**b**) of a Cu-Cr-Zr alloy after solution treatment at 920 °C and aging at 500 °C.

A copper alloy with 0.096 wt.% Cr and 0.07 wt.% Zr was produced by direct chill casting. The alloy was solution treated at 920 °C for 1 h followed by water quenching and then aged at 500 °C for 4 h. The starting microstructure was characterized by an average grain size of about 100 μm (Figure 1a) and the finely dispersed particles with a size of 9 nm (Figure 1b) and volume fraction of 6.72 × 10^−4^ as revealed through decomposition of solid solution according to the Matthiessen’s equation [13,17]. The precipitates in this alloy are mainly represented by Cr-rich particles as have been detailed elsewhere [13]. The rod samples with a cross section of 11 × 11 mm^2^ were subjected to ECAE-Conform by the so-called route A [31,32] with channel intersection at 120°. The corresponding strain after one pass was about 0.8. The used ECAE-Conform device has been detailed elsewhere [35]. The samples were subjected to 8 passes of ECAE-Conform. Then, several sets of samples after 1, 4 or 8 ECAE-Conform passes were cold rolled to different total strains of 0.6 to 4.

The developed microstructures were investigated by a Quanta 600 scanning electron microscope (SEM) equipped by an electron backscattering diffraction (EBSD) analyzer incorporating orientation imaging microscopy (OIM) with TSL OIM Analysis 6 software on the longitudinal section parallel to the plane of channel axes in the ECAE-Conform samples or that is normal to the transverse direction (TD) in the rolled samples. The OIM images were subjected to a cleanup procedure, setting 5 points for the grain dilation. The grain size was measured by the linear intercept method counting high-angle boundaries (HAB) with misorientations of θ ≥ 15° as an average of long and short intercept. The internal distortions were evaluated by means of kernel average misorientations (KAM) divided by OIM step size (h). The specimens with a gauge length of 12 mm and a cross section of 1.5 mm × 3 mm were subjected to tensile tests along the rolling direction using an Instron 5882 tensile machine at an initial strain rate of 2 × 10^−3^ s^−1^. At least two tensile specimens were tested per each data-point.

## 3. Results

### 3.1. Deformation Microstructures

**Figure 2:**
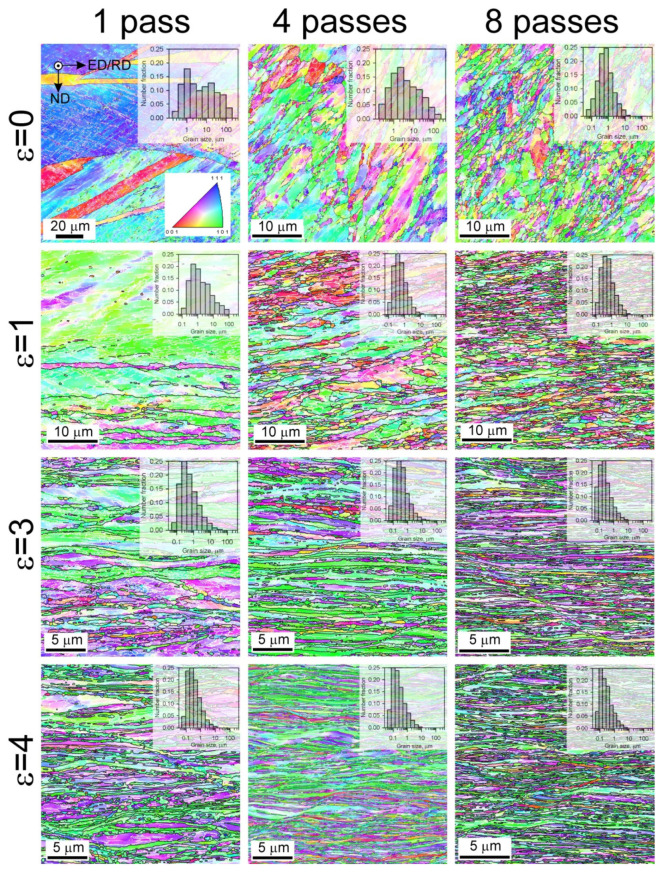
Deformation microstructures including the grain size distributions evolved in a Cu-Cr-Zr alloy during 1 to 8 passes of ECAE-Conform followed by cold rolling to a strain of 1 to 4. Colors indicate crystallographic directions along the normal direction (ND). ED and RD indicate the extrusion direction and the rolling direction, respectively. The black and white lines indicate high-angle (θ ≥ 15°) grain boundaries and low-angle (2° ≤ θ < 15°) sub-boundaries, respectively.

Typical microstructures developed in the studied Cu-Cr-Zr alloy at various stages of the present severe plastic deformation process are shown in Figure 2. ECAE-Conform results in the development of strain-induced grain boundaries arranged in deformation microbands. The number of microbands and corresponding number density of the strain-induced grain boundaries in the microbands increase with an increase in the number of ECAE-Conform passes. The frequent development of grain boundaries leads to the formation of ultrafine grains within the deformation microbands. Thus, the fraction of ultrafine grains increases with straining. Subsequent cold rolling is accompanied by the development of layered microstructure composed of interleaved bands of ultrafine grains and remnants of original grains. The evolution of the layered microstructure involves microshear bands arranged at about 30° to the rolling direction. Such microshearing provides the ultrafine grain formation in the remnants of original grains and leads to the development of lamellar-type microstructure consisting of pancaked ultrafine grains after sufficiently large rolling reductions.

**Figure 3:**
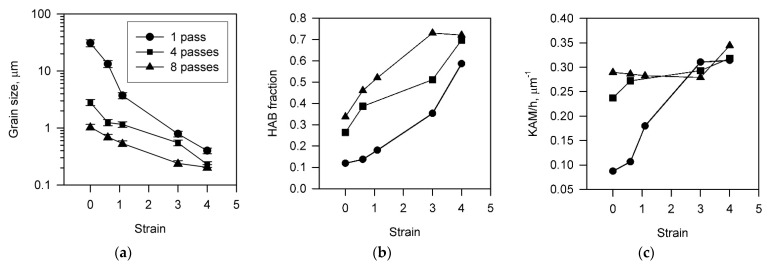
The change in the grain size (**a**), the HAB fraction (**b**), and the internal distortions as a KAM/h ratio (**c**) in a Cu-Cr-Zr alloy subjected to 1 to 8 passes of ECAE-Conform followed by cold rolling to various total strains.

The quantitative changes in the microstructure during cold rolling are represented in Figure 3 as the strain dependencies of the mean grain size, the fraction of HAB, and the internal distortions, KAM/h. The grain size gradually decreases approaching 160 nm (Figure 3a), while the fraction of HAB increases to a saturation level of approximately 0.75 (Figure 3b) during cold rolling to a total strain of 4. Similar behavior for the grain size and the HAB fraction was reported in other studies on severe plastic deformation [37,55,56,57]. Namely, the rate of grain refinement gradually decreased during deformation [57], while the HAB fraction exhibited almost linear increase with strain [56]. An increase in the number of ECAE-Conform passes promotes the evolution of the finally attainable grain size and an increase in the HAB fraction to saturation upon subsequent cold rolling. The effect of the ECAE-Conform strain on the kinetics of these microstructural changes consists in an apparent change of initial level of microstructural parameters that start to evolve during cold working. 

An increase in the internal distortions during cold rolling also significantly depends on the number of previous ECAE-Conform passes. In contrast to the grain size and the HAB fraction, the internal distortions exhibit strong dependence on the cold rolling strain in the case of 1 pass of ECAE-Conform, whereas the samples processed by 4 and 8 ECAE-Conform passes are characterized by almost the same distortion level irrespective of cold rolling strain. It is worth noting that this level of the internal distortions matches that evolved in the sample cold rolled to large strains following 1 pass of ECAE-Conform. Such behavior of internal distortions corresponds to common concept of work hardening that implies a rapid increase in the dislocation density at early deformation followed by approaching a saturation at large strains [58,59].

**Figure 4:**
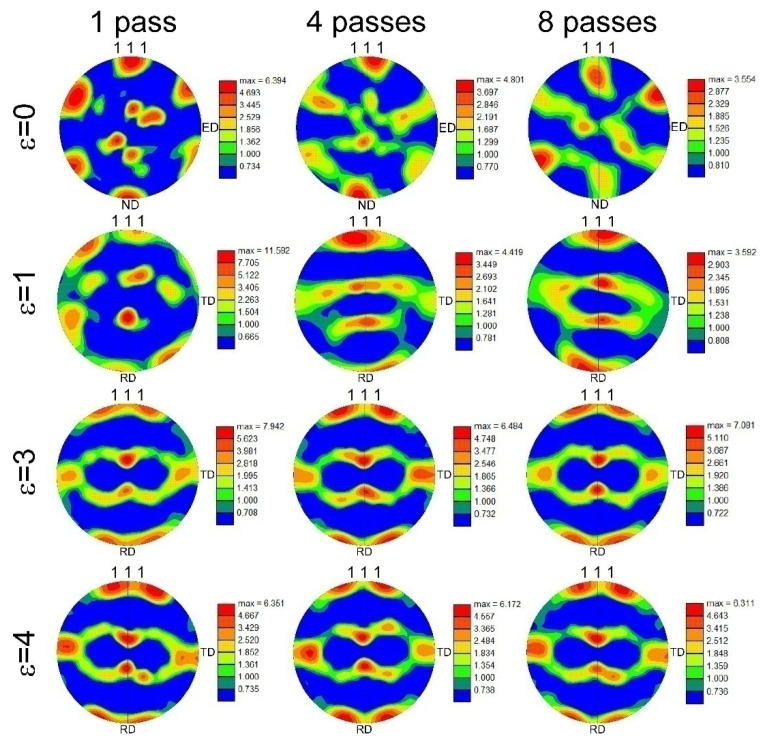
Deformation textures evolved in a Cu-Cr-Zr alloy during 1 to 8 passes of ECAE-Conform followed by cold rolling to a strain of 1 to 4. ED and ND indicate the extrusion and normal directions, respectively, for the ECAE-Conform samples according to commonly accepted approach [60]. TD and RD correspond to the transverse and rolling directions, respectively, for the rolled samples.

The deformation textures evolved in a Cu-Cr-Zr alloy by ECAE-Conform and subsequent cold rolling are shown in Figure 4. ECAE-Conform results in the development of typical pure shear texture, which has been frequently observed in various face centered cubic (fcc) metals/alloys subjected to ECAE/ECAP [60]. In spite of the relatively monotonous deformation by route A, the texture intensity does not depend remarkably on the number of ECAE-Conform passes. Probably, a maximal intensity of about 4 corresponds to a saturation level that can be attained in such copper alloys during ECAE. On the other hand, except for the sample cold rolled to a strain of 1 after 1 pass of ECAE-Conform, cold rolling is accompanied by the development of a rather strong copper texture during rolling to a strain of 1 followed by a progressive imposition of a brass texture with increasing the rolling strain, resulting in a predominance of S texture component after large strains. Such evolution of the rolling texture has been reported for fcc-metallic materials with medium stacking fault energy (SFE), when the dominant texture has changed from copper through S to brass with a decrease in SFE [61]. An exception for the sample subjected to relatively small strain is associated with rather coarse grained microstructure, the texture of which, obtained by a local OIM analysis, is significantly affected by an orientation of separate large grains. The prevalence of the brass texture component in a cold rolled precipitation strengthened copper alloy has been attributed to the development of numerous microshear bands at large strains [62]. The strong texture in the cold rolled samples results in a rather high Taylor factor of about 3.24 for tension along the rolling direction.

### 3.2. Mechanical Properties

**Figure 5:**
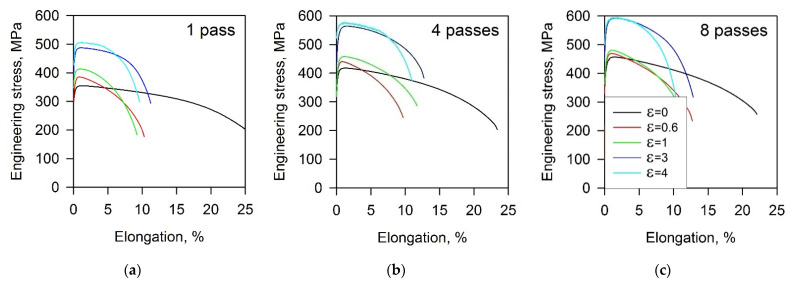
Tensile stress—elongation curves for a Cu-Cr-Zr alloy subjected to 1 (**a**), 4 (**b**), and 8 (**c**) passes of ECAE-Conform followed by cold rolling to the indicated strains (ε).

The stress–elongation curves obtained by tensile tests of the Cu-Cr-Zr alloy subjected to various strains by ECAE-Conform and cold rolling are shown in Figure 5. Cold working substantially strengthens the Cu-Cr-Zr alloy similar to other studies on severe plastic deformation [4,28,57]. The ultimate tensile strength records almost 600 MPa after 8 ECAE-Conform passes followed by cold rolling to a strain of 4. An increase in the yield strength is more pronounced as compared to the ultimate tensile strength. Hence, the yield strength tends to approach the ultimate tensile strength with an increase in total strain by ECAE-Conform followed by cold rolling. Following the yielding, the flow stresses quickly increase to their maximum, followed by gradually decreasing due to necking. The strengthening is accompanied by a degradation of plasticity, especially after cold rolling, when total elongation decreases about twofold as compared to the previous ECAE-Conform. 

**Figure 6:**
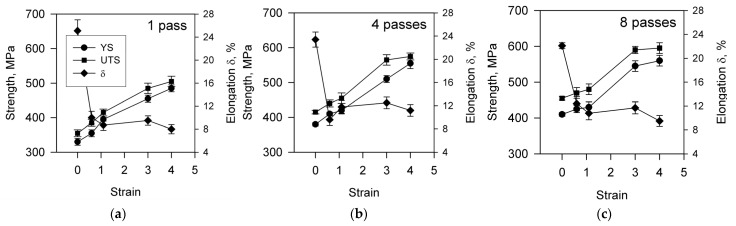
Changing the yield strength (YS), the ultimate tensile strength (UTS), and elongation (δ) of a Cu-Cr-Zr alloy after cold rolling to different strains following by 1 (**a**), 4 (**b**), and 8 (**c**) passes of ECAE-Conform.

The strain effect on the main tensile properties is represented in Figure 6. An increase in the rolling strain results in almost the same linear increase of both the yield strength and the ultimate tensile strength. An apparent decrease in the strengthening rate at large strains of 3 to 4 reflects common attenuation behavior for strain hardening of polycrystalline metallic materials [57]. In contrast, total elongation sharply drops after small rolling reductions, and then seems to stabilize at a level of 10% irrespective of rolling strain. Hence, the strengthening of the present Cu-Cr-Zr alloy during cold rolling to severely large strains is not accompanied by a remarkable decrease in plasticity. 

## 4. Discussion

### 4.1. Grain Refinement

The development of ultrafine grains in the present Cu-Cr-Zr alloy during severe plastic deformation by ECAE-Conform followed by cold rolling is closely connected with strain localization in the microshear bands. The strain-induced ultrafine grains readily evolve in the microshear bands and their intersections very similar to other studies on nanocrystalline structures produced by severe plastic deformation [57]. The progressive microshear banding increases the number of new ultrafine grains as strain increases. The grain refinement during severe plastic deformation, therefore, results from continuing to increase in the fraction of strain-induced ultrafine grains. This mechanism of microstructure evolution that is caused by continuous strain-induced reactions has been defined as continuous DRX [57]. However, the heterogeneous development of new grains associated with strain localization makes it possible using a Johnson–Mehl–Avrami–Kolmogorov equation [38], which has been recently modified to express the fraction of ultrafine grains (*F*_UFG_) as a function of total strain (ε) during continuous DRX [63].

*F*_UFG_ = 1 − exp(–*k*ε*^n^*), (1)

Here, *k* and *n* are constants depending on material and processing conditions.

Then, assuming that the new ultrafine grains with a size of *D*_UFG_ develop in an initial coarse grained microstructure, i.e., *D*_0_>>*D*_UFG_, the mean grain size (*D*_ε_) evolving upon straining has been expressed as follows [64].

*D*_ε_ = *D*_UFG_(1 − exp(–*k*ε*^n^*))^−0.5^, (2)

**Figure 7:**
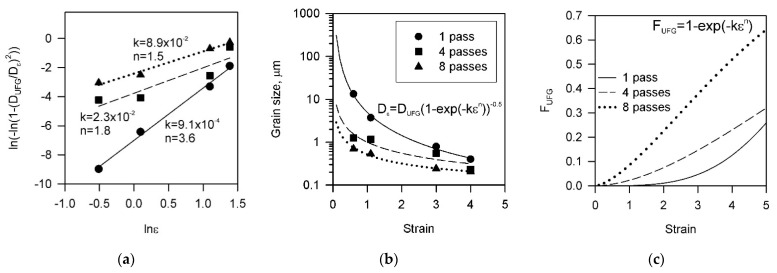
Relationships between the rolling strain and ln(–ln(1 − *D*_UFG_^2^*D*_ε_^−2^)) (**a**), the grain size (**b**), and the fraction of ultrafine grains (**c**) in a Cu-Cr-Zr alloy subjected to 1 to 8 passes of ECAE-Conform followed by cold rolling up to total strain of 4.

Taking *D*_UFG_ = 160 nm, the relationship between ln(ε) and ln(–ln(1 − *D*_UFG_^2^*D*_ε_^−2^)) is represented in Figure 7a, where the experimentally measured values are indicated by the symbols, and the lines correspond to linear fit using Equation (2). Using the values for numerical parameters obtained in Figure 7a, the grain refinement for the present Cu-Cr-Zr alloy by means of large strain cold rolling is quantitatively represented as the strain dependencies of the mean grain size and the ultrafine grain fraction in Figure 7b,c, respectively. The lines in Figure 7b correspond to values predicted by Equation (2), whereas symbols indicate experimentally measured grain sizes. Good correspondence between the predicted and measured values establishes the speculation above. In spite of apparent simplicity, the developed models (Equations (1) and (2)) adequately predict the kinetic of grain refinement during severe plastic deformation. A special advantage of the present approach is a feasibility to quantitatively take into account the effect of initial microstructure. Although the positive influence of pre-refinement of initial microstructure on the ultrafine grain evolution by subsequent severe plastic working has been suggested in previous studies [56,57], such an approach was not elaborated before.

Figure 7c suggests that the grain refinement kinetics during large strain cold rolling can be significantly accelerated by an appropriate preliminary treatment of the original microstructure. The present results indicate that the ultrafine grained microstructure with a grain size as small as 160 nm can be obtained by ordinary cold rolling. Such fine grains comprise more than 50% in the microstructure evolved by cold rolling to a total strain of 4 following ECAE-Conform, although ECAE-Conform itself does not reduce the mean grain size below 1 μm even after eight passes.

### 4.2. Strengthening by Severe Plastic Deformation

**Figure 8:**
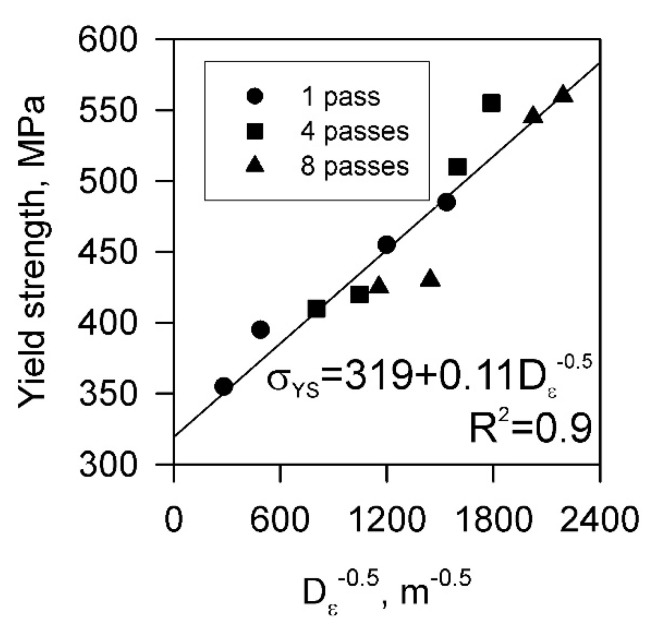
Relationship between the yield strength (*σ*_YS_) and the grain size (*D*_ε_) in a Cu-Cr-Zr alloy subjected to ECAE-Conform (1 to 8 passes) followed by cold rolling (up to total strain of 4). Note that the grain size was calculated by Equation (2).

The grain refinement in the present Cu-Cr-Zr alloy by severe plastic deformation is accompanied by significant increase in the tensile strength to almost 600 MPa. The relationship between the yield strength and the mean grain size, which was calculated by Equation (2), is presented in Figure 8. The present data obey a Hall–Petch-type plot with grain boundary strengthening factor of 0.11 MPa m^0.5^. This value lies in the range of 0.09 MPa m^0.5^ to 0.14 MPa m^0.5^ reported in other studies for copper and its alloys [39,64,65,66]. The relatively high value of 319 MPa for the first term in the Hall–Petch-type relationship in Figure 8 can be attributed to additional strengthening by dispersed particles and dislocation density. Assuming an Orowan mechanism for the dislocation–particle interaction, the dispersion strengthening (*σ*_Or_) can be calculated as follows [67].

*σ*_Or_ = 0.55 *Gb λ*^−1^ (ln(0.5*d*_P_*/*b*) + 0.7), (3)

where *G* is the shear modulus, *b* is the Burgers vector, *λ* is the edge-to-edge particle spacing, and *d*_P_* depends on the mean particle size (*d*_P_) and can be calculated as *d*_P_* = (*d*_P_^−1^ + *λ*^−1^)^−1^. Hence, the dispersion strengthening of the present alloy comprises 84 MPa. The dislocation strengthening reportedly varied from about 100 MPa to 250 MPa in pure copper and Cu-Cr-Zr alloys, respectively, subjected to severe plastic deformation [39,64,65]. Therefore, the present strengthening of about 200 MPa, which can be related to the high dislocation density evolved by ECAE-Conform, is in good agreement with previous studies.

**Figure 9:**
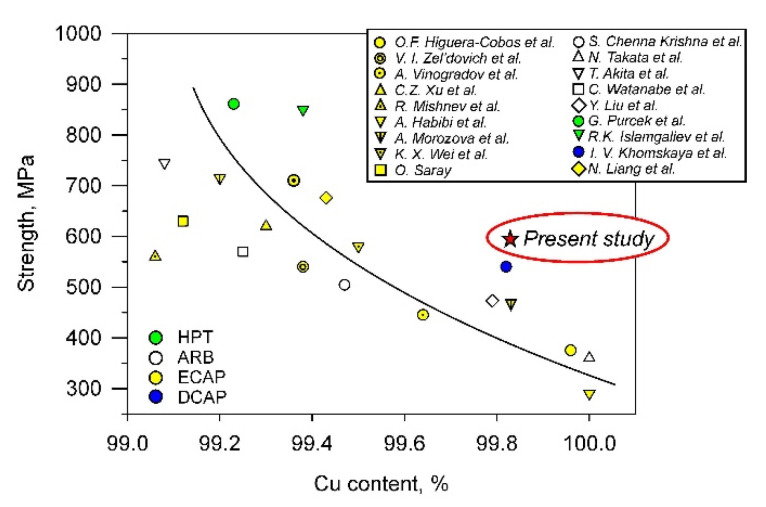
Relationship between the strength and the alloying content in copper alloys subjected to severe plastic deformation [4,5,8,29,39,68,69,70,71,72,73,74,75,76,77,78,79,80].

The beneficial effect of the severe plastic deformation as a combination of ECAE-Conform followed by cold rolling is illustrated in Figure 9, which shows the strength of the Cu-Cr-Zr alloy achieved in the present work in comparison with other published results for copper alloys with different alloying extent [4,5,8,29,39,68,69,70,71,72,73,74,75,76,77,78,79,80]. Colors in Figure 9 indicate processing method to simplify consideration. The samples processed by HPT are characterized by higher strength as compared to those after accumulative roll-bonding (ARB) or ECAP. The latter has a little advantage owing to, probably, the larger strains imposed. The most interesting samples obtained in the present study are remarkably superior in strength to others with the same alloying extent. The higher strength can be attained in copper alloys with only much larger alloying content. Note here that the strength of the present samples exceeds even that in similar copper alloy processed by dynamic channel angular pressing by means of an explosion technique [78].

The revealed regularities of the microstructure evolution in a copper alloy during severe plastic deformation, taking into account the effect of initial microstructure, i.e., pre-deformation history, combined with the observed structural dependence of the strengthening makes it possible to develop the processing methods involving severe plastic deformation in order to obtain the material with desired properties, at least, a copper alloy of certain strength. A special benefit of the present elaboration is that the present approach can be expanded to other materials and processing techniques. There is no doubt that the established relationships will promote the implementation of novel processing technologies for ultrafine grained metals/alloys with outstanding properties.

## 5. Conclusions

The regularities of microstructure evolution and the strengthening of a Cu-0.1Cr-0.1Zr alloy subjected to ECAE-Conform followed by cold rolling were studied. The main results can be summarized as follows.

An ultrafine grained microstructure could be obtained in the alloy by severe plastic deformation. An average grain size tended to approach 160 nm with an increase in the rolling reduction. The grain refinement during cold rolling could be related to total strain through the modified Johnson–Mehl–Avrami–Kolmogorov equation with fitting parameters depending on the preceding ECAE-Conform.

An increase in the number of ECAE-Conform passes significantly accelerated the development of ultrafine grained microstructure upon subsequent cold rolling. The fraction of the ultrafine grains with a size of 160 nm after cold rolling to a strain of 4 increased from 0.12 to 0.52 as the number of ECAE-Conform passes increased from one to eight.

The grain refinement during severe plastic deformation was accompanied by significant strengthening. The yield strength increased above 550 MPa after eight ECAE-Conform passes followed by cold rolling to a strain of 4. The strengthening throughout cold rolling following ECAE-Conform could be expressed by a Hall–Petch-type relationship with a grain size strengthening factor of 0.11 MPa m^0.5^.

## Data Availability

The data presented in this study are available on request from the corresponding author.
